# German Hospital Development: An Expert Continental Review

**Published:** 1914-03-14

**Authors:** 


### GERMAN HOSPITAL DEVELOPMENT.

### An Expert Continental Review.

Under the title of "Ergebnisse und Fortschritte des Krankenhauswesens" ("Incidents and Progress in the Hospital World"), the well-known firm of Gustaf Fischer, of Jena, publishes the second volume of the monumental series of books on hospitals which it began last year under the able editorship of Professor Dietrich, who is one of the chief consulting experts on hospitals in Germany, and as such an expert adviser of the Ministry of the Interior. This second volume is edited by Professor Grober, chief of the medical clinic at Jena, and is a most interesting and well-printed book, with 166 illustrations, issued at a net price of 23s.

The object of the series is to give a yearly record of hospital development and progress in such a form as to be easily referred to by hospital workers and architects. It has been the editor's intention to make the books thoroughly authoritative; the plans given are drawn to scale, and careful estimates and measurements are given in each case. Hence the articles are not journalistic condensations barely describing the novelties, but expert criticisms and useful comparisons with already well-known standards. They are more or less on a common basis, which considerably simplifies the business of anyone wishing to find a suitable criterion for whatever work he is interested in. Naturally such a design demands from those responsible for carrying it out the greatest discrimination in judging what new buildings, apparatus, methods, or items are worthy to be accepted as standards. On the Continent, and more especially in Germany, progress in hospital work is so rapid that it is not to be wondered at if there is sometimes an innovation which is really a regrettable retrograde movement. Even in wide ranging essentials is such a retrograde movement to be witnessed.

### Hospital Costliness: A German View.

Some of us, for example, hold that the modern tendency, so curiously popular with modern German hospital architects, of centralising different departments in one block is unfortunate and mistaken. But it is worth while to consider carefully what are the reasons which have led to popularising this tendency, strikingly exhibited in the new Charité buildings at Berlin. Economy of space, forced upon the designers through the rapidly increasing value of land in the large cities, is undoubtedly one of the reasons; but there are also others. This very subject is admirably dealt with by Dr. Krohne in Professor Grober's new book. His article is entitled "The Increasing Costliness of our Modern Hospital System: a Consideration of Causes and Suggestions for Combating Them." It is a most thoughtful and instructive article, which well repays careful reading. Dr. Krohne shows the disadvantages, financial and otherwise, which this increasing costliness brings in its train to rate-supported hospitals, and the no less severe (though, since they are not borne directly by the ratepayers but are felt almost vicariously, less generally appreciated) disadvantages entailed by this increase upon the community generally. He argues that the increasing expenditure is in some ways compensated by increased efficiency, but this argument does not hold all along the line. The cost of site values, of bricklaying, of timber-work, of stone-work, of drainage, and of machinery generally has gone up out of all proportion to the ultimate benefits which the institutions derive from first-class handiwork. The chapter is one of the most readable and interesting in the book; and, although its tone is at times highly pessimistic, the final conclusions are such that everyone who has studied hospital progress during the past ten years will agree with them.

Herr Karl Sudhoff, of Leipzig, contributes the first chapter, which is a historical sketch of mediæval hospitals—one of those thoughtful and all too rare contributions to the history of an interesting subject which we have hitherto looked for only in *Janus* or similar antiquarian journals. Architect Boethke deals with mediæval French hospitals; Dr. von Merkel, of Nuremberg, with "Hospital Doctors and Superintendents." The fourth chapter is that of Dr. Krohne already alluded to. The fifth is a critical consideration of the new hospitals in Baden Baden by Professors von Hofmeister, Weintraud, and Grober. This gives full plans, details of construction and equipment, and accounts of administration and management; it is altogether a highly informative article, which has the added merit of being expert criticism on a wide comparative basis.

### The Berlin Ambulance System.

The sixth chapter is devoted to a critical account of the Berlin ambulance system, and is from the pen of Councillor Dr. Gottstein, of Charlottenburg, an acknowledged authority on this subject. It is particularly interesting on account of the historical details given and also for the discussion of the communal value of the Berlin system. A short, but informative and ably written, chapter is by Dr. Werner on "Tropical Hospitals," which gives interesting information regarding the construction of new buildings in hot climates, such as, for instance, the large new Government hospital at Dar-es-salam. The next chapter is a discussion of hospital problems in large cities, written by Dr. Gottstein. This and the succeeding chapter—the *rôle* of the scientifically trained, skilled woman worker in hospitals, by Herr Wolff—are concerned with such gigantic problems that it is unfair to the authors to review them in a paragraph; they should be read in their entirety, with a pencil at hand to make marginal annotations for careful consideration at leisure. A concise chapter on "The Equipment of the Modern Hospital," written by

Professor Grober, follows. It must be distinctly stated that all these chapters deal, not so much with old premisses or matters already discussed in text-books, but largely with new points that have arisen or come into prominence during the past year. Take, for example, the recent enormous development in certain methods of treatment—notably adjunct methods such as radium and electro-therapeutics.

THE CONSTRUCTION OF RADIUM STORES.

The hospital worker, and the architect as well, wish to know something about the equipment which is needed for installing successfully these new methods. No text-book as yet gives detailed information which can be relied upon; while monographs on the methods themselves are written by medical men who have no means of considering the points which, from a constructional point of view, especially interest the hospital worker. We saw very much the same thing when *x*-ray treatment was winning popularity in the hospitals. Some of the earlier *x*-ray rooms were miserable affairs. Dr. Grober's article is an attempt to collate what has been written and said so as to make the whole assimilable for the constructionist; for that reason his chapter is a very valuable contribution to the literature.

The third part of the book is devoted to construction pure and simple. A general article on building developments during the year is contributed by Architect Boethke, who gives plans which are always well chosen so as to illustrate points that are really worth noting. This section contains several highly technical chapters, of interest more especially to architects, but of value also to medical men and to hospital workers generally. We refer, more particularly, to Architect May's excellent article on special departments in the infants' clinic; Boethke's on the machine and boiler house in large hospitals; and Schwickart's on new methods in planning and constructing laundries and disinfecting units. Further interesting contributions are: a distinctly original article by Dr. Dosquet on "Hospitals as Curative Agents through Ventilation and Lighting"; Dr. Stoevesandt's chapter on "The Air-Cure Hospital for Men at Bremen"—a description of a very original sanatorium whose plans are likely to be hotly criticised by various schools; and May's further chapter on the new buildings of the Kaiserin Victoria Haus at Berlin. Dr. Dietrich contributes an ably written article on administrative problems; he give copies of the various rules and regulations for new German and American hospitals, and discusses whatever new point they raise. A special article is devoted to the new regulations for the medical and nursing staff at the Mount Sinai Hospital at New York. Finally the editor gives a general *résumé* of hospital progress during the past year, while Professor Roth contributes a very full and really valuable bibliography that should be of great use to those who refer to it. An admirable index closes a volume which for catholicity of information, wealth of criticism, and excellence of text would be hard to rival.

